# Protection of stromal cell-derived factor-1 SDF-1/CXCL12 against proteases yields improved skin wound healing

**DOI:** 10.3389/fimmu.2024.1359497

**Published:** 2024-08-02

**Authors:** Rafaela Vaz Sousa Pereira, Mostafa EzEldeen, Estefania Ugarte-Berzal, Jennifer Vandooren, Erik Martens, Mieke Gouwy, Eva Ganseman, Jo Van Damme, Patrick Matthys, Jan Jeroen Vranckx, Paul Proost, Ghislain Opdenakker

**Affiliations:** ^1^ Laboratory of Immunobiology, Department of Microbiology, Immunology and Transplantation, Rega Institute for Medical Research, KU Leuven, Leuven, Belgium; ^2^ Department of Imaging and Pathology, OMFS-IMPATH Research Group KU Leuven and Oral and Maxillofacial Surgery, University Hospitals Leuven, Leuven, Belgium; ^3^ Department of Oral Health Sciences, KU Leuven and Pediatric Dentistry and Special Dental Care, University Hospitals Leuven, Leuven, Belgium; ^4^ Laboratory of Molecular Immunology, Department of Microbiology, Immunology and Transplantation, Rega Institute for Medical Research, KU Leuven, Leuven, Belgium; ^5^ Department of Development & Regeneration & Department of Plastic & Reconstructive Surgery, University Hospitals Leuven, KU Leuven, Leuven, Belgium

**Keywords:** SDF-1, CXCL12, proteolysis, chemokine, COAM, wound healing

## Abstract

SDF-1/CXCL12 is a unique chemotactic factor with multiple functions on various types of precursor cells, all carrying the cognate receptor CXCR4. Whereas individual biological functions of SDF-1/CXCL12 have been well documented, practical applications in medicine are insufficiently studied. This is explained by the complex multifunctional biology of SDF-1 with systemic and local effects, critical dependence of SDF-1 activity on aminoterminal proteolytic processing and limited knowledge of applicable modulators of its activity. We here present new insights into modulation of SDF-1 activity *in vitro* and *in vivo* by a macromolecular compound, chlorite-oxidized oxyamylose (COAM). COAM prevented the proteolytic inactivation of SDF-1 by two inflammation-associated proteases: matrix metalloproteinase-9/MMP-9 and dipeptidylpeptidase IV/DPPIV/CD26. The inhibition of proteolytic inactivation was functionally measured by receptor-mediated effects, including intracellular calcium mobilization, ERK1/2 phosphorylation, receptor internalization and chemotaxis of CXCR4-positive cells. Protection of SDF-1/CXCL12 against proteolysis was dependent on electrostatic COAM-SDF-1 interactions. By *in vivo* experiments in mice, we showed that the combination of COAM with SDF-1 delivered through physiological fibrin hydrogel had beneficial effect for the healing of skin wounds. Collectively, we show that COAM protects SDF-1 from proteolytic inactivation, maintaining SDF-1 biological activities. Thus, protection from proteolysis by COAM represents a therapeutic strategy to prolong SDF-1 bioavailability for wound healing applications.

## 1 Introduction

Stromal cell-derived factor-1 (SDF-1) or CXCL12 is a homeostatic and multifunctional chemokine, involved in different physiological processes, such as embryogenesis, hematopoiesis and angiogenesis (1). SDF-1 biological functions are mediated mostly by a G protein-coupled receptor CXCR4, when the N-terminal region of SDF-1 binds to and activates this receptor (2). CXCR4 is expressed on multiple cell types, including leukocytes, hematopoietic stem cells and other progenitor cells. Like most chemokines, SDF-1 also binds glycosaminoglycans and this interaction is important for its *in vivo* function(s) (3).

SDF-1 has beneficial or detrimental effects according to organ or disease entities (4). For instance, a beneficial role of SDF-1 has been described for tissue repair, whereas pathological effects are shown in cancer, arthritis and pulmonary diseases (5–9). Hence, both delivery and inhibition of SDF-1 have been suggested as therapeutic approaches (10, 11).

In skin wound healing, SDF-1 is involved in the recruitment of endothelial progenitor cells, providing a significant contribution to wound neovascularization and re-epithelialization (12, 13). Beneficial effects of SDF-1 have also been described on wound macrophages (14). However, SDF-1 is susceptible to proteolysis and inactivation by many proteases, including dipeptidyl peptidase IV (DPPIV)/CD26, neutrophil elastase, Cathepsin G and different metalloproteinases (MMPs) (15–18). Due to the highly proteolytic environment in wounds, which is even more exacerbated in chronic wounds, SDF-1 bioavailability becomes a challenge in environments with tissue damage, including all kinds of traumata and skin wounds. To solve this problem for skin wound healing, studies rely on daily SDF-1 injections or on more complex approaches, such as the on-site production of extra SDF-1 by transformed lactic acid bacteria or lentiviral vectors, or by injections of genetically modified mesenchymal stem cells expressing SDF-1 (12, 14, 19–21). Sustained delivery of SDF-1 through liposomes or biomaterials has also been investigated (22–24). All the named approaches aim to increase the exogenous production and local delivery of SDF-1 and only one study tackles the problem of proteolysis of endogenous or exogenous SDF-1 by CD26 in the context of skin wound healing (14). Here, we investigated the inhibition of proteolysis of SDF-1. Because SDF-1 is amino-terminally truncated and its activity is killed by several proteases of different catalytic classes, we searched for broad-spectrum and rather general proteolysis inhibition, because this would widen the application range.

Chlorite oxidized oxyamylose (COAM) is a polyanionic polysaccharide derivative with immunomodulatory effects, e.g. by interference with the chemokine system. Through its capacity to bind to different chemokines, COAM acts as a mimetic of glycosaminoglycans and induces the recruitment of innate immune cells (25–27). Aside from its mechanism of inducing and binding endogenous chemokines, another advantage of COAM is the prolonged action when parenterally or locally applied (28).

In this study, we investigated the ability of COAM to protect SDF-1 against proteolytic degradation and we explored the use of COAM-SDF-1 association as a potential strategy to increase SDF-1 bioavailability in a model of mouse skin wound healing.

## 2 Materials and methods

### 2.1 Proteolytic processing of SDF-1

Recombinant human SDF-1 (300 ng) from Peprotech (UK), was incubated with 5 µl of neutrophil degranulates (NDs, containing mixtures of neutrophil proteases) or with purified active human MMP-9 (recombinant human MMP-9 produced, purified and activated as described in (29)) at a molar ratio of 1/100 (protease/SDF-1), unless indicated otherwise, in a buffer containing 150 mM NaCl, 10 mM CaCl_2_, 30 mM Tris and 0.01% Tween-20 (pH 7.4), for 4 h at 37°C. For proteolysis experiments by CD26 (R&D Systems, UK), SDF-1 was incubated with a molar ratio of 1/200 (CD26/SDF-1) in 25 mM Tris buffer (pH 8), for 2 h at 37°C. In experiments with COAM, SDF-1 was pre-incubated with COAM (mass excess; as indicated) for 1 h before proteolysis by NDs, MMP-9 or CD26. Neutrophil degranulates were collected from human blood granulocytes stimulated with N-formylmethionyl-leucyl-phenylalanine (fMLP) from Sigma (USA), as previously described (30).

### 2.2 COAM synthesis

COAM synthesis involved two-step oxidation of amylose, as previously described (31). Briefly, the first step oxidation of amylose by sodium periodate is followed by a second oxidation step by sodium chlorite. These reactions yield COAM, a compound with multiple carboxyl groups. COAM batches prepared were free of endotoxin and of protein contaminants, determined by Limulus amebocyte lysate test and SDS-PAGE electrophoresis followed by Coomassie Brilliant Blue or silver staining, respectively (32).

### 2.3 SDS-PAGE and Edman sequencing

After proteolysis, SDF-1 samples were separated in Novex 16% tricine gels (Thermo Fisher Scientific, USA) and stained with the SilverQuest Silver Staining Kit. Digital images of the gel were then captured. Cleaved SDF-1 forms were visualized as stained protein bands, migrating at lower molecular weights than the original intact SDF-1. For Edman sequencing, proteins blotted onto PVDF membranes were stained with Coomassie Brilliant Blue, excised and analyzed with a PPSQ-51A protein sequencer (Shimadzu, Japan).

### 2.4 Western blot

For Western blot analysis of SDF-1 proteolysis, SDF-1 samples were separated in Novex 16% tricine gels. For Western blot analysis of SDF-1 signaling responses in THP-1 cell lysates, samples were separated in Novex 4-20% Tris-Glycine gels (Thermo Fisher Scientific). Lysate proteins were then transferred onto Immun-Blot FL PVDF membranes (Bio-rad, USA) and blocked for 1 h with Intercept Blocking Buffer (LI-COR Biosciences, USA). For proteolysis analysis, membranes were incubated overnight at 4°C with a mouse monoclonal antibody against SDF-1 (clone K15C, 1:2000, Merck Millipore, USA) and with a rabbit polyclonal antibody against SDF-1 (1:1000, Cell Signaling Technology, USA). For signaling analysis, membranes were incubated with a rabbit antibody against p-ERK1/2 Thr202/Tyr204 (1:1000, Cell Signaling Technology) and with a mouse antibody against ERK1/2 (1:2000, Cell Signaling Technology). After washing, the blot membranes were incubated for 1h at room temperature with the secondary antibodies: anti-mouse IgG 800CW and anti-rabbit IgG 680RD for proteolysis analysis or anti-rabbit IgG 800CW and anti-mouse IgG 680RD for signaling analysis (1:20000, LI-COR Biosciences). After sufficient washes, the membranes were scanned on an Odyssey Imaging System (LI-COR Biosciences). Relative quantification of intact (uncut) SDF-1 (in green) normalized to total SDF-1 (in red) and of p-ERK1/2 normalized to total ERK1/2 were performed with the use of Image Studio Lite Software (LI-COR Biosciences).

### 2.5 Cell culture

Human monocytic THP-1 cells were cultured in RPMI1640 medium supplemented with glutamine (GlutaMAX, Gibco), 10% FBS and 3% sodium bicarbonate. For signaling experiments, THP-1 cells were serum-starved in RPMI medium without phenol red for 24 h. Cells were stimulated with 30 ng/mL SDF-1 forms (with or without prior proteolysis) for 2 minutes and cell lysates were obtained with the use of RIPA buffer and protease inhibitors.

### 2.6 Calcium-mobilization assay

THP-1 cells (10×10^6^/mL) were stained with 2.5 µM of the fluorescent dye Fura-2AM (Invitrogen, USA) in HEPES buffered saline (HBS) containing 0.1% bovine serum albumin (BSA) and 0.01% Ploronic-F127 (Sigma), for 30 minutes at 37°C. After staining, cells were washed twice with HBS and resuspended at 1x10^6^ cells/mL. For each individual test condition, 1.8×10^6^ cells were preheated for 10 min at 37°C prior stimulation with 30 ng/mL SDF-1 and SDF-1 cleaved by MMP-9 or CD26 in the presence or absence of COAM. At 120 seconds of recording, buffer control (Vehicle) or SDF-1 forms were added. Fura-2 fluorescence was measured on a LS50 B luminescence spectrometer (Perkin Elmer, USA) at 510nm after excitation at 340 and 380nm. Intracellular Ca^2+^ concentrations ([Ca^2+^]i) were calculated using the Grynkiewicz equation (33). R_max_ and R_min_ values were determined via the treatment of cells with 50 µM digitonin and 10 mM EGTA (Sigma) in 20 mM Tris buffer (pH 8.5; Merck, Germany), respectively. Results were analyzed with WinLab32 software (Perkin Elmer, USA). Fluorescence intensities of unloaded cells (not treated with Fura-2AM) were used to correct the results for auto-fluorescence. To quantify the peak of intracellular calcium concentration, the baseline measurement was subtracted from the maximum value of the curve (*i.e.* peak level).

### 2.7 Receptor internalization

For receptor internalization experiments, THP-1 cells were stimulated with 100 ng/mL SDF-1 and SDF-1 cleaved by MMP-9 in the presence or absence of COAM for 15 minutes. Next, the cells were washed with ice-cold PBS and incubated for 15 min with the Fc-receptor-blocking antibodies anti-CD16/anti-CD32 (BD Biosciences Pharmingen) and with a Zombie Aqua™ viability dye (BioLegend, USA) for 15 min at 4°C. After washing with cold PBS + 2% FBS, surface receptors were stained with phycoerythrin (PE)-conjugated anti-CXCR4 clone QA18A64 (Biolegend) for 30 min at 4°C. Cells were washed and analyzed on a Flow Cytometer BD LSR Fortessa X20 with DIVA software. The results were further analyzed with the FlowJo software (BD). CXCR4 mean fluorescence intensity (MFI) was calculated and the results were expressed as percentage relative to the unstimulated condition (100% CXCR4 expression).

### 2.8 Chemotaxis assays

Chemotactic responses of SDF-1 were measured *in vitro* using a multiscreen disposable device with a 96-well filter (5 µm pore size) and a 96-well receiver plate. THP-1 cell suspensions (100 µl in the filter plate at cell concentration of 3.5x10^6^ cells/mL) and test samples (150 µl in the receiver plate at concentration of 30 ng/mL) were diluted in RPMI medium (without phenol red) supplemented with 0.1% BSA. After 3 h of migration at 37°C, the upper 96-well filter plate was removed and the cells in the lower receiver plate were quantified with the luminescence ATP detection assay system (ATPlite) (Perkin Elmer). ATP concentrations were proportional to the cell numbers. The chemotaxis index was calculated by dividing the luminescence value of the test sample by the luminescence value of the control buffer. Results of test samples were normalized relatively to SDF-1 control samples, considered as 100% THP-1 cell migration. Chemotatic responses into SDF-1-containing fibrin hydrogels were also measured *in vitro* in the presence and absence of COAM with the use of multiscreen plates. Hydrogels (50 μl fibrin hydrogel with SDF-1 at 1 μg/mL) were placed at the bottom of the well (receiver plate). After 4 h of migration, hydrogels were digested with Nattokinase (50 FU/mL) and migrated cells were counted using a Fortessa Flow Cytometer.

### 2.9 MMP-9 activity assay

MMP-9 activity tests were performed as previously described (34). Briefly, activated MMP-9 was incubated with COAM. Next, dye-quenched gelatin (DQ™-gelatin, Invitrogen) was added at a final concentration of 2.5 µg/mL and the increase in fluorescence over time was measured with the CLARIOstar microplate reader (BMG Labtech).

### 2.10 CD26 activity assay

To measure the CD26 activity, the chromogenic substrate Gly-Pro p-nitroanilide p-toluenesulfonate (GP-p-NA) was used. CD26 was incubated with COAM and mixed with 500 µM Gly-Pro-pNA in 25 mM Tris Buffer (pH 8.0). CD26 activity was determined kinetically (every 5 minutes for 2h at 37°C) by measuring the enzymatic conversion of GP-p-NA to p-NA (absorbance at 405 nm).

### 2.11 Hydrogel composition and preparation

Fibrin hydrogels were prepared as described previously (35, 36). Briefly, plasminogen-depleted human fibrinogen (Enzyme Research Laboratories, USA) was prepared in fibrinogen buffer (20 mM HEPES and 150 mM NaCl). Thrombin (Sigma) and factor XIII (Fibrogammin, CSL Behring, Germany) were prepared in thrombin buffer (20 mM HEPES, 150 mM NaCl, 40 mM CaCl_2_ and 0.1% BSA) and kept in a water bath at 37°C for 30 min to activate factor XIII to factor XIIIa. Fibrinogen and thrombin components were mixed to prepare hydrogels with 3.5 mg/ml fibrinogen, 0.1 U/ml thrombin and 0.1 U/ml factor XIII. SDF-1, COAM or SDF-1/COAM were incorporated into fibrinogen component preparation before mixing with thrombin components.

### 2.12 Wound model

Full-thickness excisional wounds were induced on the back of C57BL/6J mice. All procedures were conducted in accordance with the regulations of the European Union (directive 2010/63/EU) and were approved by the local Animal Ethics Committee of KU Leuven (License numbers P270/2015, P128/2019). Briefly, mice were anesthetized with ketamine (100 mg/kg) and xylazine (10 mg/kg) and the hair was removed using a shaver and hair removal cream. The skin was lifted at the mid-dorsal line, folded and punched through with a 4mm disposable sterile biopsy punch, creating one wound on each side of the dorsal midline. The procedure was repeated one more time, generating 4 wounds per animal. Fibrin hydrogels containing SDF-1, COAM or SDF-1/COAM were applied locally in a volume of 25 µl per wound. The control mice were treated only with fibrin hydrogels (Vehicle). SDF-1 was used at 1 µg and COAM at 100 µg (mass excess of 100x) in the hydrogels applied to the wounds. Digital images of the wounds were captured and the wound sizes were quantified with the software ImageJ. Wound healing analysis was expressed as percent healed in relation to wound diameter on day 0 and expressed as area under the curve (AUC).

### 2.13 Statistical analysis

Data analysis was done using GraphPad Prism 9.1.2. Data were shown as mean ± SEM. A normality test was applied and all data followed a normal distribution. Therefore, the Student´s two-tailed unpaired t-test was used when analyzing two groups. Otherwise, the one-way ANOVA test was used. A two-way ANOVA test was used for the analysis of wound repair over time. Statistical significance was determined at p < 0.05.

## 3 Results

### 3.1 COAM protects SDF-1 from proteolysis by MMPs and other neutrophil proteases

**Figure 1:**
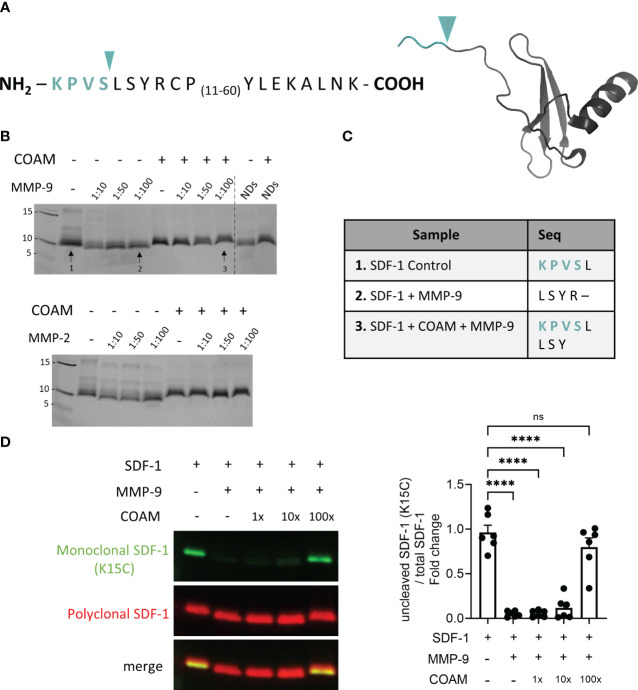
COAM protects SDF-1 from proteolysis by MMPs. **(A)** Amino acid sequence and crystal structure of human recombinant SDF-1. Blue arrowhead and amino acid sequence in blue color indicate the cleavage site by MMPs and the residues which are removed in the cleaved form of SDF-1, respectively. **(B)** Human recombinant SDF-1 was incubated with COAM (100x mass excess) prior to incubation with different ratios of active MMP-9, MMP-2 or neutrophil degranulates (NDs). SDS-PAGE gel followed by silver staining illustrating SDF-1 cleavage (lower molecular weight bands). Arrows numbers 1, 2 and 3 indicate the conditions used for sequencing analysis. **(C)** Edman sequencing data showing the detected amino acids of the N-terminal region of SDF-1 under the three conditions analyzed. **(D)** SDF-1 was incubated with different ratios of COAM (mass excess) prior to MMP-9 incubation. Western blot analysis of the uncut form of SDF-1 with a monoclonal antibody (in green) and total forms, including cleaved and uncut forms with a polyclonal antibody (in red). One representative Western blot is shown and the summary of the quantification of experiments (n=6) is documented by the histogram. Quantification of uncut form normalized to total SDF-1 and expressed as relative values to control condition (uncut SDF-1). Each data point represents an independent experiment. **** p <0.0001. Ns, not significant. Results were statistically analyzed by One-way ANOVA with Dunnett’s multiple comparisons test.

SDF-1 is a substrate for many proteases, including MMPs and serine proteases. Studied MMPs cleave SDF-1 between the 4^th^ (serine/Ser/S) and 5^th^ amino acid (leucine/Leu/L) in the N-terminal region and inactivate SDF-1 (15) (**Figure 1A**). Using different ratios of protease *versus* substrate we confirmed the cleavage of SDF-1 by MMP-9 and MMP-2, illustrated by the lower molecular protein band corresponding to the cleaved SDF-1 form (**Figure 1B**). Interestingly, when SDF-1 was incubated in the presence of COAM (100x mass excess *versus* SDF-1) prior to proteolysis by MMPs, the reduction in molecular weight as a result of SDF-1 cleavage was not observed, suggesting that COAM protected SDF-1 from proteolysis by MMP-9 and MMP-2 (**Figure 1B**). Similar results were obtained when proteolysis was induced by neutrophil degranulates (NDs) (**Figure 1B**), containing proteases with different catalytic mechanisms (37). The latter experiments suggested that COAM protected SDF-1 against proteolysis in an unselective way. N-terminal sequencing analysis of SDF-1 incubated with MMP-9 resulted in the sequence LSYR, confirming the expected cleaved site by MMP-9 between the serine and leucine amino acids (**Figure 1C**; sample 2). In samples, in which SDF-1 was incubated with COAM before the cleavage by MMP-9, both KPVSL and LSY sequences were detected, which corresponded to uncut and cleaved forms, respectively (**Figure 1C**; sample 3). In the latter specific sample, the most predominant form detected was the uncut form, since higher percentages of the amino acids detected in each cycle corresponded to the uncut SDF-1 form (71% Edman cycle 1 corresponding to Lys, 87% cycle 2 corresponding to Pro and 95% cycle 3 corresponding to Val).

For complementation, we performed additional experiments in which we used a monoclonal antibody able to detect only the intact form of SDF-1 (visualized with the use of green fluorescence), together with a polyclonal antibody able to recognize both cleaved and uncut forms (indicated by red fluorescence). Marginal detection of the uncut form was observed when SDF-1 was incubated with MMP-9, confirming the high efficiency of MMP-9 in cleaving SDF-1. In contrast, pre-incubation with COAM at 100x excess resulted in significant protection against SDF-1 cleavage (around 80% of uncut form remaining) (**Figure 1D**). At the intermediate COAM concentration tested (10x) a protection of around 12% against degradation was observed. Together, these data confirmed that COAM indeed protected SDF-1 against degradation by MMP-9 and other proteases.

### 3.2 COAM protects SDF-1 from proteolytic inactivation preserving biological activity

**Figure 2:**
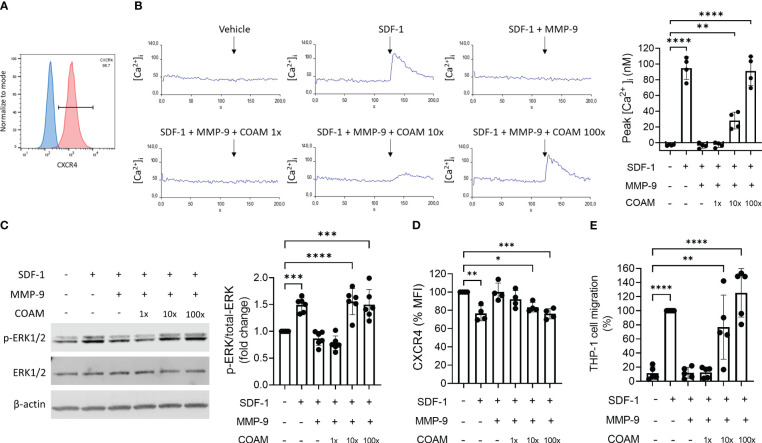
COAM protects SDF-1 from inactivation by MMP-9 and preserves its biological activity. **(A)** Cell surface CXCR4 expression in THP-1 cells by Flow Cytometry. Percentage of CXCR4-positive population is indicated. Blue and red histograms correspond to unstained and CXCR4 stained cells, respectively. **(B)** Real-time changes of intracellular calcium levels in function of time. At 120 seconds of recording, THP-1 cells were stimulated with different SDF-1 forms (after MMP-9 proteolysis). The primary data of one representative example are shown. Peaks of intracellular calcium concentrations were quantified and the results of all experiments (n=4) were summarized in the histogram. **(C)** Western blot analysis of phospho-ERK1/2, total ERK1/2 and β-actin expression levels in THP-1 cells stimulated with SDF-1 forms (after MMP-9 proteolysis) for 2 minutes. Quantification of phospho-ERK1/2 normalized to total ERK1/2 and expressed as relative values to control condition (unstimulated). One representative Western blot is shown and the summary of the quantification of experiments (n=6) is documented by the histogram. **(D)** Cell surface expression of CXCR4 determined by Mean Fluorescence Intensity (MFI) in THP-1 cells after 15 minutes of stimulation with SDF-1 forms. Data are expressed as relative values to unstimulated condition, representing steady-state quantities of cell surface CXCR4 (100% CXCR4) (n=4). **(E)** THP-1 cell migration towards SDF-1 forms (after MMP-9 proteolysis). Data expressed as percentage of migration relative to SDF-1 control group (100% migration) (n=5). Each data point represents an independent experiment. *p < 0.05 **p < 0.01 ***p < 0.001 and ****p <0.0001. Results were statistically analyzed by One-way ANOVA with Dunnett’s multiple comparisons test.

SDF-1 binds to the cell surface receptor CXCR4 and triggers activation of diverse signaling pathways, to promote CXCR4-mediated chemotaxis. Therefore, it was relevant to study whether SDF-1 (after proteolysis by MMP-9 in the absence or presence of COAM) was biologically active and able to activate CXCR4 or was devoid of activity. To test this, we performed functional assays using THP-1 cells, a monocytic cell line that expresses the CXCR4 receptor (38) (**Figure 2A**). First, we evaluated intracellular calcium mobilization ([Ca^2+^]i) following THP-1 cell stimulation with SDF-1 forms. The clear intracellular calcium signal seen upon stimulation with intact SDF-1 was completely abolished by MMP-9 proteolysis of SDF-1 (**Figure 2B**). Pre-incubation with COAM 100x maintained the ability of SDF-1 to induce calcium signals at similar levels as observed by the uncut form. Moreover, COAM 10x pre-incubation was sufficient to induce a significant SDF-1 mediated calcium signal (**Figure 2B**). The phosphorylation of the kinase ERK1/2 was also investigated in response to SDF-1 stimulation. Whereas stimulation with uncut SDF-1 resulted in increased ERK1/2 phosphorylation, SDF-1 cleaved by MMP-9 failed to trigger ERK1/2 activation. Interestingly, both COAM 10x and 100x treatments were able to induce ERK1/2 phosphorylation at comparable levels to the uncut SDF-1 (**Figure 2C**).

Another response that is induced by SDF-1 binding to CXCR4 is the recruitment of β-arrestin, which can lead to receptor internalization and enhancement of MAPK pathway activation. Therefore, we also tested the ability of SDF-1 forms to induce CXCR4 internalization. SDF-1 forms incubated with COAM 100x in the presence of proteases promoted CXCR4 internalization at similar levels to the uncut form (25% of receptor internalization), whereas cleaved SDF-1 had no effect on surface receptor expression (**Figure 2D**). Similar to the effect observed in calcium signaling experiments, COAM 10x induced less but still significant receptor internalization.

Lastly, SDF-1-induced chemotaxis was evaluated and, as expected, the ability of SDF-1 to induce cell migration was completely inhibited with the cleaved SDF-1 form and entirely restored with the COAM 100x pre-incubated form (**Figure 2E**). Once more, the intermediate concentration of COAM (10x) induced intermediate but significant cell migration.

Together, these data indicate that COAM protects SDF-1 against processing and inactivation by MMP-9, maintaining SDF-1 biological activity.

### 3.3 COAM also protects SDF-1 against CD26 proteolytic inactivation

**Figure 3:**
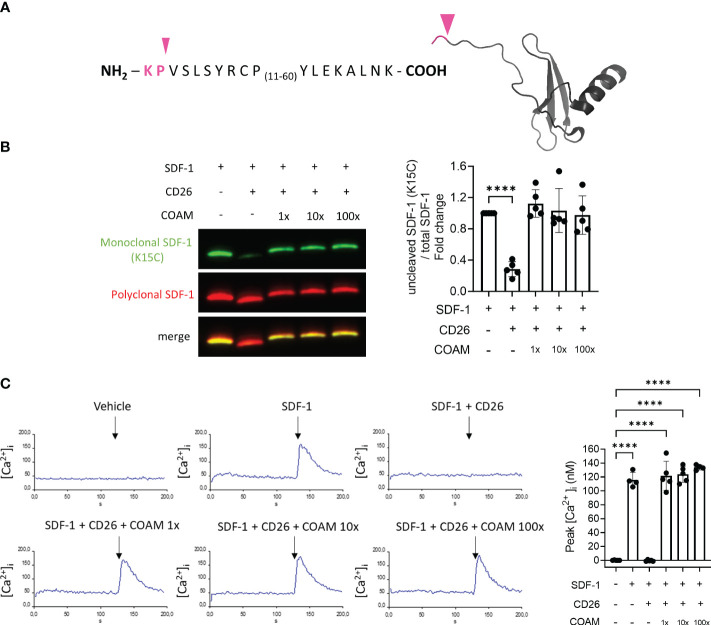
COAM protects SDF-1 against CD26 proteolytic inactivation. **(A)** Amino acid sequence of human recombinant SDF-1. Pink arrowhead and pink sequence indicate the cleavage site by CD26 and the residues which are removed in the cleaved form of SDF-1, respectively. **(B)** SDF-1 was incubated with different ratios of COAM (mass excess) prior to CD26 incubation. Western blot analysis of the uncut form of SDF-1 with a monoclonal antibody (in green) and total forms, including cleaved and uncut forms with a polyclonal antibody (in red). Quantification (n=5) of uncut form normalized to total SDF-1 and expressed as relative values to control condition (uncut SDF-1). **(C)** Real-time changes of intracellular calcium levels in function of time. At 120 seconds of recording, THP-1 cells were stimulated with different SDF-1 forms (after CD26 proteolysis). Peaks of intracellular calcium concentrations were quantified and the collected data summarized in the histogram (n=5). Each data point represents an independent experiment. ****p <0.0001. Results were statistically analyzed by One-way ANOVA with Dunnett’s multiple comparisons test.

Dipeptidyl peptidase IV (DPPIV) or CD26 is a serine protease known to cleave SDF-1 between the 2^nd^ (proline/Pro/P) and 3^rd^ amino acid (valine/Val/V) in the amino terminus (**Figure 3A**). Considering the fact that CD26 is an important inflammatory regulator of SDF-1 activity (39), we investigated whether the proteolytic protection of SDF-1 by COAM is also applicable to CD26 proteolysis. Surprisingly, we observed that COAM inhibited SDF-1 degradation by CD26 more efficiently than the protection observed for MMP-9. A complete protection against proteolysis was already seen upon pre-incubation with an equal mass of COAM (**Figure 3B**). Similar levels of protection were observed with COAM 10x and 100x mass excess pre-incubation. We confirmed this finding through the functional evaluation in the intracellular calcium signal assay in response to SDF-1 stimulations and reinforced once more that SDF-1 bound to COAM is still active and able to induce cellular responses at similar levels to the intact form (**Figure 3C**).

### 3.4 SDF-1 proteolytic protection by COAM is dependent on COAM-SDF-1 electrostatic interactions

**Figure 4:**
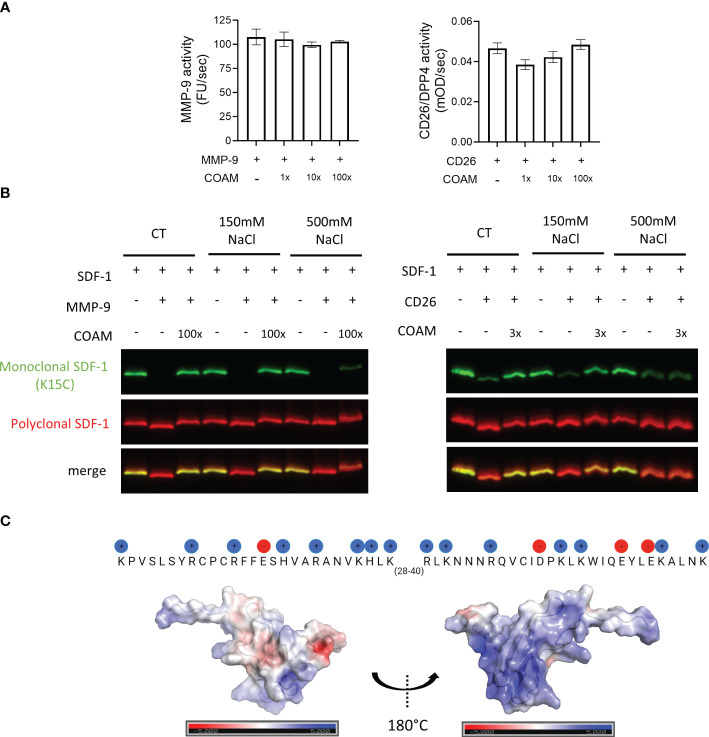
SDF-1 proteolytic protection by COAM is dependent on COAM-SDF-1 electrostatic interactions. **(A)** MMP-9 and CD26 activity in the presence or absence of COAM measured using DQ™-gelatin and Gly-Pro-pNA as substrate, respectively (n=3-4). **(B)** SDF-1 was incubated with COAM and NaCl prior to incubation with the indicated proteases. Western blot analysis of the uncut form of SDF-1 with a monoclonal antibody (in green) and total forms, including cleaved and uncut forms with a polyclonal antibody (in red). **(C)** The amino-acid sequence of SDF-1, highlighting the positively (blue) and negatively (red) charged amino acids, and the surface positions of these charges onto the folded protein (3D structure).

To investigate if COAM could have a direct effect on the catalytic activities of the used proteases or if other mechanisms may be involved, we performed MMP-9 and CD26 enzymatic activity assays. No major changes in MMP-9 or CD26 enzymatic activities were detected in the presence of COAM (**Figure 4A**), thus confirming that COAM did not interfere directly with enzyme activities. These findings supported the idea that direct binding of COAM to SDF-1 and protection against proteolysis are involved in the effects observed above. Due to the negatively charged nature of COAM and the positive charges in SDF-1, we hypothesized that the interaction between these two components is direct and dependent on electrostatic charges. To confirm this, we compared proteolysis experiments in the presence and absence of an excess of NaCl to neutralize the electrostatic interactions. While at physiological salt concentrations (150mM NaCl) the SDF-1 proteolytic protection by COAM was clearly observed, an excess of NaCl (500mM NaCl) inhibited the ability of COAM to protect SDF-1 from MMP-9 and CD26 degradation (**Figure 4B**). Importantly, such high NaCl concentrations did not interfere with the capacity of MMP-9 and CD26 to cleave SDF-1. To illustrate possible electrostatic interactions of the negatively charged COAM with SDF-1, the amino-acid sequence of SDF-1, highlighting the positively (blue) and negatively (red) charged amino acids, and the surface positions of these charges onto the folded protein (3D structure) are shown in **Figure 4C**. Together, these data suggest that the beneficial effect of COAM is associated with its capacity to bind to SDF-1 and protect it against proteolysis in a charge-dependent way.

### 3.5 COAM-SDF-1 as a potential therapeutic application for wound healing

**Figure 5:**
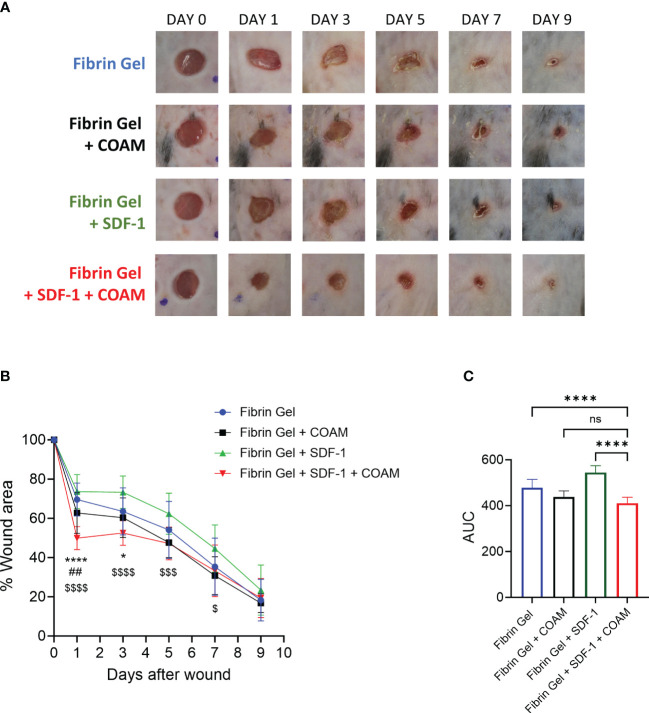
SDF-1/COAM combination as treatment for wound repair. Wounds were treated with COAM, SDF-1 or SDF-1/COAM incorporated into fibrin hydrogels. **(A)** Representative digital images of wounds up to 9 days after wounding. **(B)** Wound healing was expressed as percentage of wound area compared to day 0. *p<0.05, ****p<0.0001 Fibrin Gel + SDF-1 + COAM vs Fibrin Gel. ##p<0.01 Fibrin Gel + SDF-1 + COAM vs Fibrin Gel + COAM and $p<0.05, $$$p<0.001, $$$$p<0.001 Fibrin Gel + SDF-1 + COAM vs Fibrin Gel + SDF-1. Result was statistically analyzed by Two-Way ANOVA test with Tukey’s multiple comparisons test. **(C)** Wound healing expressed as area under the curve (AUC). ****p<0.0001. Result was statistically analyzed by One-Way ANOVA test with Tukey’s multiple comparisons test. Ns, not significant. n≥10 mice per group in two independent experiments.

In various *in vivo* studies SDF-1 had beneficial effects on wound healing (12–14). Considering the proteolytic inactivation of SDF-1 by proteases present under inflammatory conditions (*e.g.* neutrophil proteases, MMP-9, CD26) and our finding of protection of SDF-1 against proteolysis with the use of COAM, we hypothesized that the combination of COAM with SDF-1 might be a good application to deliver SDF-1 into wounds and to avoid the fast degradation by several proteases present in the wound environment. To investigate this, we delivered SDF-1 and COAM incorporated into a fibrin hydrogel and analyzed the wound repair over time. We compared the effect of SDF-1/COAM combination treatment with control (Fibrin Gel), COAM (Fibrin Gel + COAM) and SDF-1 (Fibrin Gel + SDF-1). We observed that wounds treated with the combination SDF-1/COAM had a significant reduction in wound size on days 1 and 3 when compared to wounds of the control group (Fibrin Gel) (**Figures 5A, B**). When compared to wounds treated with COAM (Fibrin Gel + COAM), the SDF-1/COAM combination showed wound size reduction only at day 1 post-injury. The difference between wounds treated with SDF-1/COAM combination and wounds treated only with SDF-1 (Fibrin Gel + SDF-1) extended until 7 days post-wounding. Analysis of the areas under the curve (AUC) better illustrated these differences and highlighted the positive effect of SDF-1/COAM combination when compared to Fibrin Gel and Fibrin Gel + SDF-1 groups (**Figure 5C**).

## 4 Discussion

SDF-1 is a homeostatic chemokine with beneficial effects for tissue repair. SDF-1 activity is dependent on interaction with its receptors and this interaction is abolished by NH_2_-terminal truncation of SDF-1 (1). Several proteases are involved in SDF-1 inactivation, dampening the use of SDF-1 as a therapeutic approach in several applications. Therefore, we investigated here a strategy to increase SDF-1 availability for wound healing purposes. We showed that COAM, a polysaccharide derivative, protects SDF-1 from degradation by several proteases, including MMP-9 and CD26. Most importantly, several biological activities of SDF-1 were retained once it was bound to COAM. Moreover, the COAM/SDF-1 association accelerated the healing of skin wounds and may represent a therapeutic strategy to assist wound repair.

COAM shares some common characteristics with glycosaminoglycans (GAGs), which include a linear structure, negative charges (due to carboxyl groups in the case of COAM) and capacity to bind to chemokines. Although COAM can bind to several chemokines, some selectivity exists in the chemokine binding capacity of COAM. For example, while COAM binds with high affinity to CXCL1, CXCL2, CXCL10 and CXCL11, no interaction was detected with the chemokines CCL2, CCL3 and CCL4 (25). In addition to the binding of all the chemokines previously described by Li et al. (25), COAM also binds to SDF-1 and the affinity of COAM to SDF-1 was higher than to heparan sulfate (unpublished data; EzEldeen M. et al., in preparation 2024). We have previously shown that administration of COAM increases the recruitment of monocytes/macrophages to the wounds, probably due to the binding of COAM to endogenous chemokines (27).

SDF-1 possesses a GAG-binding region that comprises a collection of amino acids with a positive charge. This specific area, referred to as the BBXB domain, is characterized by the presence of basic amino acids denoted as ‘B’ (representing positively charged residues) and any other residues indicated as ‘X’ (40). In the SDF-1 sequence, this domain corresponds to KHLK (position 24-27). Aside from the GAG-binding domain, additional amino acids with positive charges and the spatial arrangement of these positively charged amino acids within the 3-dimensional structure of SDF-1 molecule play a crucial role in its capacity to bind to GAGs (1, 41). Here we showed that, similar to GAGs, the binding of SDF-1 to COAM is also dependent on electrostatic interactions and this interaction is essential for the anti-proteolytic protection effect of COAM. Although the precise mapping of the interaction between SDF-1 and COAM is not known, this interaction seems to be of non-covalent nature. Additional experiments, for instance with the use of recombinant SDF-1 mutants, will be needed to map the SDF-1 residues interacting with COAM. CXCR4, as the SDF-1 receptor, is a target of proteinases (17). In view of the net negative charge of the ligand-binding N-terminal part of CXCR4, it is unlikely that COAM will protect against CXCR4 proteolysis.

Although in the present study we focused on two purified inflammation-associated proteases (the metalloproteinase MMP-9 and the serine protease CD26), in view of our findings using neutrophil degranulates (NDs) we suggest that COAM-mediated protection of SDF-1 proteolysis covers a diverse group of proteases. Serine proteases, such as neutrophil elastase and cathepsin G, are known to cleave SDF-1 in the N-terminal region and this cleavage leads to its inactivation (1). We have shown previously that serine proteases were the main proteases present in neutrophil degranulates responsible to cleave IL-7 (42). SDF-1 interaction with GAGs, specifically with heparin and heparan sulphate, has also been shown to prevent the cleavage of SDF-1 by CD26 (43). In contrast, McQuibban et al. described that association of SDF-1 with different GAGs did not inhibit the cleavage by MMP-2 (15).

For the wound healing application, as detailed here, we delivered COAM and SDF-1 through a physiological fibrin scaffold hydrogel. In a previous study, we showed that the fibrin hydrogel on its own induced considerable changes in the wound environment towards a faster wound repair, and therefore, it represents an optimal vehicle for the delivery of therapeutic molecules into wounds (36). Importantly, the inclusion of COAM does not alter the microstructure nor the stiffness of fibrin hydrogels (35). In view of the observed beneficial effect of COAM/SDF-1 association for wound repair with a single treatment/dose, we believe that the inhibition of SDF-1-cleaving proteases COAM observed *in vitro* is also happening *in vivo*, probably by extending the SDF-1 bioavailability in the wound environment. This comes as an advantage compared to other studies in which daily treatments with SDF-1 are necessary to overcome the fast degradation of SDF-1 (19, 20). Additionally, more complex approaches have been investigated to obtain a sustainable delivery of SDF-1 into the wounds (12, 14, 21).

In their review, Zhao and colleagues summarize various strategies to incorporate SDF-1 into biomaterials for regenerative medicine applications (44). According to these authors, SDF-1/scaffold bonding strategies can be classified as physical and chemical immobilization, but importantly, the premature degradation of SDF-1 should also be prevented (44). Incorporation of SDF-1 into hydrogels, such as alginate and antioxidant thermoresponsive hydrogels, was shown to induce sustained release of SDF-1 with positive effects for wound healing (24, 45). These studies, however, do not address the proteolysis problem faced by SDF-1 susceptibility to several proteases. In our approach, the three beneficial elements of the above-mentioned reviewed strategies were combined. First, the incorporation of COAM into fibrin hydrogel provides a physical immobilization important for local action. By *in vitro* chemotaxis assays with THP-1 cells into fibrin hydrogels, we determined that the effect of SDF-1 was maintained in the presence of COAM, illustrating the biocompatibility of all three compounds (data not shown). Thereby, the binding of COAM to SDF-1 represents a chemical immobilization of the ligand, without any loss of critical biological activities of SDF-1, such as chemotaxis. Third, the inhibition of SDF-1 degradation by proteases, present in wounds and inflammation, is a completely new insight at the biochemical level. This inhibitory activity of COAM against several classes of proteinases cleaving SDF-1 implies that the local bioavailability of SDF-1 is prolonged and that daily injections of SDF-1 are not needed anymore for a clinical effect, because a single treatment resulted in beneficial outcomes *versus* controls. Similar to our approach, a GAG-based hydrogel made with heparin was prepared to deliver SDF-1 and it was shown to induce a sustained delivery of SDF-1, attracting circulating pro-angiogenic cells in a model of subcutaneous implantation of hydrogel to study *in vivo* angiogenesis (46). In this study, the authors assumed that SDF-1 proteolysis protection will be obtained by the heparin present in the scaffold. However, as mentioned previously, while SDF-1 interaction with GAGs has been shown to prevent the cleavage of SDF-1 by CD26, it failed in protection against cleavage by MMP-2 (15, 43). Additionally, Seeger et al. suggested that heparin also binds to CXCR4 and disrupts SDF-1/CXCR4 signaling on bone marrow-derived mononuclear cells (47). Similarly, soluble GAGs at high concentrations reduced the capacity of CXCL9, CXCL10 and CXC11 to induce calcium mobilization through CXCR3 (48). In our study, the mass excess of COAM (100x) did not impair SDF-1 interaction with the receptor (measured by calcium influx, ERK signaling and CXCR4 internalization), suggesting that there is no competition of COAM for the binding of SDF-1 with its receptor nor that the formation of complexes COAM-SDF-1 inhibited the receptor activation.

Although we have not yet investigated the mechanism behind the beneficial effect of COAM/SDF-1 treatment in skin wounds, we believe that, on a theoretical basis, one element is the involvement of increased recruitment of endothelial progenitor cells (EPCs) to the wound. EPCs express high levels of CXCR4 and are recruited to injured tissue in response to SDF-1, where they play an important role in new vessels formation (49). Effects of SDF-1 on wound macrophages are also possible as mechanism. Vågesjö et al. showed that the delivery of SDF-1 by lactic bacteria increase the population of TGF-β^+^ macrophages in skin wounds (14). Full-thickness skin wounds in aged mice fully regenerate whereas in mice, younger than 1 month, SDF-1 mediates scar formation (50). These results imply that the effects of SDF-1 may change during ontogeny. Although we did not observe any qualitative differences in scar formation between our experimental groups, it remains a point for further exploration. As a detail, we used mice of 10 weeks old and applied exogenous SDF-1 in animal hosts with normal endogenous SDF-1 production.

In conclusion, we discovered that COAM, a negatively charged amylose-derivative, bound to SDF-1 through electrostatic interactions and prevented proteolysis of SDF-1 and inactivation by several proteases, including those in neutrophil degranulates. The association of COAM with SDF-1 promoted skin wound healing above control levels in mice and this association may represent a potential therapeutic strategy with beneficial outcome for tissue repair.

## Data availability statement

The raw data supporting the conclusions of this article will be made available by the authors, without undue reservation.

## Ethics statement

The animal study was approved by Animal Ethics Committee of KU Leuven (License numbers P270/2015, P128/2019). The study was conducted in accordance with the local legislation and institutional requirements.

## Author contributions

RVSP: Conceptualization, Data curation, Formal analysis, Investigation, Methodology, Project administration, Software, Validation, Writing – original draft, Writing – review & editing. ME: Data curation, Methodology, Writing – review & editing. EU-B: Data curation, Investigation, Methodology, Writing – review & editing. JV: Data curation, Investigation, Methodology, Writing – review & editing. EM: Data curation, Formal analysis, Investigation, Methodology, Writing – review & editing. MG: Data curation, Formal analysis, Investigation, Methodology, Writing – review & editing. EG: Data curation, Formal analysis, Investigation, Methodology, Writing – review & editing. JVD: Funding acquisition, Resources, Writing – review & editing. PM: Supervision, Writing – review & editing. JJV: Funding acquisition, Supervision, Writing – review & editing. PP: Funding acquisition, Resources, Writing – review & editing. GO: Conceptualization, Funding acquisition, Investigation, Resources, Supervision, Writing – original draft, Writing – review & editing.
